# The availability of essential medicines in public healthcare facilities in Afghanistan: navigating sociopolitical and geographical challenges

**DOI:** 10.1093/heapol/czae121

**Published:** 2024-12-19

**Authors:** Margo van Gurp, Sandra Alba, Maida Ammiwala, Sayed Rahim Arab, Sayed Murtaza Sadaat, Fazelrabie Hanifi, Sohrab Safi, Nasratullah Ansari, Maiza Campos-Ponce, Maarten Olivier Kok

**Affiliations:** Health, KIT Royal Tropical Institute, Mauritskade 64, Amsterdam 1092 AD, The Netherlands; Health, KIT Royal Tropical Institute, Mauritskade 64, Amsterdam 1092 AD, The Netherlands; Health, KIT Royal Tropical Institute, Mauritskade 64, Amsterdam 1092 AD, The Netherlands; Particip, Kabul, Afghanistan; Particip, Kabul, Afghanistan; Particip, Kabul, Afghanistan; Particip, Kabul, Afghanistan; Independent Global Health Consultant; Department of Health Sciences, Faculty of Science, Vrije Universiteit, Boelelaan 1105, Amsterdam 1081 HV, The Netherlands; Department of Health Sciences, Faculty of Science, Vrije Universiteit, Boelelaan 1105, Amsterdam 1081 HV, The Netherlands; Erasmus School of Health Policy and Management, Erasmus University, Burgemeester Oudlaan 50, Rotterdam 3062 PA, The Netherlands

**Keywords:** availability of medicines, universal health coverage, contracting out healthcare, Afghanistan

## Abstract

During the past two decades, the Afghan government, along with the international community, has developed a system aimed at improving access to essential healthcare services under Afghanistan’s challenging sociopolitical and geographical circumstances. In 31 provinces, nonstate actors competed for fixed-term contracts to implement a predefined package of healthcare services. In three provinces, the government organized the provision of healthcare services. An independent third party monitored service provision, including access to medicines. This study examines the availability of essential medicines in Afghanistan’s public healthcare facilities and how this is shaped by sociopolitical challenges, geographical barriers, and the organization of the healthcare system. Between March and July 2021, enumerators collected data at 885 healthcare facilities across Afghanistan. For our analysis, we combined data on medicine availability and the functioning of the health system with publicly available information about geographical and sociopolitical factors, including security incidents. Using regression analysis, we identified facility-, district-, and provincial-level factors related to medicine availability in public healthcare facilities. On average, 70% of 31 selected essential medicines were available in 2021. The availability of medicines varied significantly between provinces and was considerably higher in those where services were contracted out to nonstate actors (*n* = 31; 91%) compared to provinces where service provision was organized by the government (*n* = 3; 9%). The most important drivers of variation in medicine availability included geographical barriers, securing and allocating funds at the provincial level, and organizing and sustaining physical capacity at the facility level. Insecurity was not a key factor driving variation in medicine availability. Despite the sociopolitical challenges in 2021, the availability of essential medicines in public healthcare facilities was relatively high. The results suggest that decentralized procurement of medicines by nonstate actors and timely payment of funds contribute to medicine availability. Strategies to improve medicine availability should target hard-to-reach areas and lower-level facilities.

Key messages

- Stock outs of essential medicines pose a serious risk to patients’ health outcomes due to missed, interrupted, or delayed treatment and can expose them to substandard or falsified medicines from private and unregulated outlets.
- In 2021, the average availability of 31 selected essential medicines was high in Afghanistan (70%) despite its sociopolitical challenges. Geographical barriers, securing and allocating funds at the provincial level, and sustaining physical capacity at the facility level drive variations in medicine availability in public healthcare facilities.
- Medicine availability was higher in provinces where healthcare services were contracted out to nonstate providers, but adequate budgets and timely disbursement of funds are required to maintain a proper supply of medical resources.
- Strategies to improve medicine availability should target facilities with lower service levels and facilities in hard-to-reach and low-income areas.

## Introduction

The availability of essential medicines in public healthcare facilities is crucial for the functioning of the health system and for achieving universal health coverage. The World Health Organization defines essential medicines as ‘those that satisfy the priority needs of the population’. Stock outs of essential medicines in public facilities pose serious risks to patient’s health due to delayed, interrupted, and missed treatments. Limited availability in public facilities drives patients to seek medications from private and unregulated outlets, increasing the risk of encountering costly, expired, and falsified medications. Additionally, a lack of medicines can contribute to antimicrobial resistance if appropriate regimens cannot be adhered to (Kranzer and Ford 2011, Koomen et al. 2019, Olaniran et al. 2022).

Previous studies have shown that medicine availability is influenced by geographical and sociopolitical factors and the organization of the health and pharmaceutical systems (Armstrong-Hough et al. 2020). Geographical and political factors like rurality, distance to distribution points, insecurity, and conflict are known barriers to ensuring continuous medicine availability (Moye-Holz et al. 2017, Ranghieri et al. 2017, Seidman and Atun 2017). Conversely, appropriate facility management and local governance (policies, information flow, and financial planning) could improve medicine availability in healthcare facilities (Moye-Holz et al. 2017, Seidman and Atun 2017).

In Afghanistan, the geographical and sociopolitical context makes health service delivery challenging. The country is mountainous and prone to various natural hazards, including earthquakes, flooding, and heavy snowfall. For this reason, and in combination with poor infrastructure, many parts of the country are often poorly accessible (Ranghieri et al. 2017). In addition to these geographical challenges, the country has been dealing with conflict for four decades. Between 2001 and 2021, the Taliban held control of a varying number of districts, limiting the central government’s access and control of the country (Mapping Taliban Control in Afghanistan, 2017). Under those challenging circumstances, the Afghan Ministry of Public Health (MoPH) collaborated with international donors such as the US Agency for International Development (USAID), the World Bank (WB), and the European Commission and tried to develop a functional health system that guarantees access to essential healthcare services and medicines nationwide (Box 1) (Newbrander et al. 2014).

After the previous Taliban regime ended in 2001, the country’s health system was in a dire state. The MoPH and international donors designed a system whereby health services were contracted out to nonstate service providers in 31 out of 34 provinces (Palmer et al. 2006). In the remaining three provinces, healthcare services were provided by the public sector through provincial health directorates, which would contract in management and technical support from nonstate providers (Arur et al. 2010). Contracting out healthcare services was considered the most efficient way to scale up service delivery rapidly (Palmer et al. 2006, Frost et al. 2016). Under this model, service providers compete for fixed-term contracts to implement a predefined package of essential healthcare services for primary healthcare centres and hospitals in a province (Newbrander et al. 2014). Their performance and compliance with guidelines are verified through an extensive third-party monitoring system (Alba et al. 2023). Despite considerable improvements in healthcare access since the introduction of this package of public healthcare services, challenges in healthcare quality and efficiency have been persistent. Therefore, the MoPH and the WB introduced a pay-for-performance component in 2019, in which a negotiated percentage of the contract value of service providers was paid based on performance indicators (Samad et al. 2023).

The designers of this system anticipated that the combination of service provision by a nonstate actor with ties to the community and understanding of the local context, competitive and temporary contracting, and extensive monitoring by a third party would make it possible to provide healthcare services under Afghanistan’s challenging geographical and political circumstances. It provides service providers with the flexibility and incentives to manage resources effectively; this includes flexibility in the procurement of medicines to reduce stock outs in healthcare facilities (Salehi et al. 2018). This level of flexibility offered certain advantages in a country like Afghanistan, with considerable variation in security and geographic accessibility. A procurement approach that works for a highly urbanized population centre like Kabul may not work for a mountainous or otherwise hard-to-reach province that is inaccessible for parts of the year. On the other hand, different procurement practices can give rise to fragmentation, potentially leading to disparities in medicine availability and creating pockets of under-resourced areas.

While Afghanistan has made significant progress in providing access to healthcare services for its population, it remains unclear to what extent contracting nonstate actors has ensured the availability of essential medicines in its challenging sociopolitical and geographical circumstances. Gaining insight into the factors driving variations in medicine availability is crucial for developing policies that improve access, reduce inequalities, and enhance our understanding of strategies to provide healthcare services in fragile and conflict-affected settings.

This study aims to assess the availability of essential medicines in Afghanistan’s public healthcare facilities and analyse the political, geographical, and health system factors driving variation in medicine availability. Data were collected in 2021, just months before the Taliban took control of the government. Enumerators visited 885 healthcare facilities to conduct a national healthcare facility assessment and document the availability of essential medicines. We analysed the availability of 31 essential medicines and explored the relationship between availability, local insecurity, geographical barriers, and the organization of the health and pharmaceutical systems.

## Materials and methods

### Study setting and design

This cross-sectional study uses data from the Balanced Scorecard (BSC) of primary healthcare facilities conducted in 2021. The BSC was an annual healthcare facility assessment implemented from 2004 to 2021 to monitor the availability and quality of public healthcare services across all 34 provinces in Afghanistan. The results of the BSC were used to monitor the implementation of public healthcare services in Afghanistan and to improve the quality of service delivery in the country (Box 1). The questionnaires used for this assessment, including the list of medicines to be included, were determined by key stakeholders, including non-governmental organizations (NGOs), MoPH, and development partners (Peters et al. 2007).

The selection of variables was guided by their relevance to the Afghan health and pharmaceutical systems. Variables were categorized into eight dimensions: sociopolitical factors, geographical barriers, managing pharmaceutical product supply, local governance and stewardship, securing and allocating funds, organizing and sustaining physical capacity, and guaranteeing product safety. We selected relevant variables at the provincial, district, and facility levels to emphasize the multilevel structure of the health system, whereby national policies and local components of the health system shape medicine availability on the ground.

Box 1. **System Enhancing for Health Actions in Transition (SEHAT) and Sehatmandi, the backbone of Afghanistan’s public healthcare sector**

In Afghanistan, implementation of healthcare services is contracted out to nonstate service providers in 31 provinces and through public provision by provincial health directorates in three other provinces (Panjsher, Parwan, and Kapisa). Service providers and provincial health directorates were responsible for implementing essential healthcare services outlined in the Basic Package of Health Services (BPHS). The BPHS was designed in 2002 to address the most pressing health issues of underserved populations, focusing on maternal and child healthcare services. Financing came from the WB, the USAID, and the European Union (EU). Each donor initially targeted specific provinces, but their efforts were brought together in 2013 under the ‘SEHAT’ programme and continued in 2019 as Sehatmandi (Sehatmandi Project|MoPH). Under SEHAT and Sehatmandi, service providers periodically tendered for fixed-term contracts per province through a bidding process. In Panjsher, Parwan, and Kapisa, services were implemented by the government, but managerial staff and technical assistance were contracted in from nonstate providers (also referred to as the strengthening mechanism) (Arur et al. 2010). Service providers and public health directorates were subjected to third-party monitoring of their performance at the provincial level. Contracts were managed and reviewed by the MoPH (Salehi et al. 2018, Alba et al. 2023).

### Data collection and sources

A total of 892 healthcare facilities were selected following a stratified random sampling approach from a list of active primary care facilities under the Sehatmandi project (Box 1). Within each province, a random sample of 5 subhealth centres (SHCs), 15 basic health centres (BHCs), 5 comprehensive health centres (CHCs), and 2 district hospitals (DHs) was drawn. If a province had an insufficient number of a particular type of healthcare facility, additional facilities were sampled from a lower level. Seven of the 892 sampled healthcare facilities could not be reached due to roadblocks or insecurity. Data from the remaining 885 healthcare facilities were collected between March and July 2021. Within each healthcare facility, a systematic random sample of 10 clients was observed and interviewed (five patients under 5 years old and five patients over 5 years old). In addition, a systematic random sample of 4 healthcare workers was interviewed per healthcare facility and 10 per DH.

District-level indicators related to conflict and insecurity were extracted from the Armed Conflict Location and Event Data Project and the *Long War Journal* (Raleigh et al. 2010; Mapping Taliban Control in Afghanistan, 2017). Provincial-level data on out-of-pocket expenditures and contract information were extracted from the 2018 Afghanistan Health Survey and semiannual performance reports (Afghanistan Health Survey (AHS) 2018; Sehatmandi Provincial Semi-Annual Performance Review, 2019).

### Variables

The primary outcome of this study was the percentage of medicines out of a total of 31 essential medicines that were continuously available in the healthcare facility in the past 30 days (see Supplementary Table S2 for the list of medicines). The BSC includes 54 essential medicines. However, medicines for malaria, leishmaniasis, tuberculosis, and vaccines were excluded as these are not expected to be available across the country and all facility types. Table 1 provides an overview of all covariates for each dimension and shows whether they are measured at the facility, district, or provincial level. Supplementary Table S1 provides an overview of all covariates considered during the analysis; not all were included in the final model. Supplementary Table S4 provides an overview of missing data per variable.

**Table 1.** Definition of covariates included in statistical analysis by dimension

Dimension	Variable	Definition
Security	Control of district^a^	Whether district is under government control, Taliban control, or contested on 9 July 2021.
	Taliban influence^b^	Whether the province is under no, moderate, medium, or high Taliban influence.
	Security incidents^a^	Number of security incidents in a district from January until July 2021, including abduction/forced disappearance, air/drone strike, armed clash, attack, grenade, remote explosive/landmine/improvised explosive device, shelling/artillery/missile attack, and suicide bomb.
	Civilian fatalities^a^	Number of civilians killed or injured resulting directly or indirectly from conflict-related violence between January and July 2021 in a district.
Geographical barriers	Travel time to the provincial centre	Travel time from the healthcare facility to the provincial centre by car is <4, between 4 and 8, or >8 hours.
	Travel time to the nearest ring road	Average travel time in the district to the nearest ring road (in hours).
	Altitude	Healthcare facility located on the sea level (<500 m), low altitude (500–2000 m), moderate altitude (2000–3000 m), or high altitude (>3000 m).
	Risk of natural hazards^a^	The healthcare facility is located in a district at high risk of natural hazards, such as avalanches, earthquakes, landslides, flooding, or drought.
Local governance and stewardship	National Monitoring Checklist	The facility applied a national monitoring checklist at least once in the past 12 months.
	Supervision visits	The healthcare facility received a supervision visit from the provincial health department or NGO last month.
Organizing and sustaining physical and human resources	Facility type	Type of healthcare facility (SHC, BHC, CHC, or DH).
	Salary payment up-to-date	At least one healthcare worker reports that salary payment is not up-to-date.
	Healthcare worker-to-patient ratio	Number of healthcare workers per 100 patients.
	Closed healthcare facility	The healthcare facility was temporarily closed in the month preceding the survey.
Managing pharmaceutical product supply	Drug inventory	The healthcare facility has a drug inventory.
	Up-to-date facility status report	The healthcare facility has an up-to-date facility status report.
	Patient satisfaction with ease of obtaining medicine	At least 80% of clients are satisfied with the ease of obtaining medicines.
	Pharmacy management training	At least one healthcare worker trained in pharmacy management in the past 12 months.
	Patient satisfaction with availability of medicines^b^	The average level of satisfaction with the availability of medicines in the province.
Securing and allocating funds	Lump sum^b^	Percentage of the provincial contract value as a lump sum as opposed to performance-based.
	Budget per capita^b^	Contracting in, <10 USD per capita, 10–20 USD per capita, and 20–30 USD/capita.
	Out-of-pocket expenditure on drugs^b^	Share of provincial average of out-of-pocket expenditure for out-patient visits spent on drugs.
	Poverty^b^	The percentage of people with the lowest wealth quintile.
Guaranteeing product safety	Electricity supply	Electricity is available all day, most of the day, a few hours per day, or not at all.
	Appropriate prescription practices	There is evidence that healthcare workers give out medicines without proper instructions.
	Pharmacy stock	Pharmacy stock (including thermometer and exhaust fan) is present and functional.

a Measured at the district level.

b Measured at the provincial level.

### Analytical approaches

We employed four analytical approaches to assess how the health and pharmaceutical systems and sociopolitical and geographic dimensions affect the availability of medicines: (I) a descriptive analysis to assess the level variation in the availability of medicines, (ii) a regression analysis to assess associations between covariates and medicine availability, (iii) an analysis of the explained variance (*R*^2^) to provide insights into how much each dimension contributes to the overall model, and (IV) an analysis of spatial autocorrelation to ensure that the model accounts for spatial dependencies. All analyses accounted for stratification at the provincial level and differential selection probabilities using design weights. The analyses were performed using Stata version 15 and GeoDa version 1.8.

#### Descriptive and regression analyses

As a first step, we performed a descriptive analysis to assess the availability of medicines on a national level and the extent to which variation in the availability of medicines exists between facilities, provinces, and medicine categories. Averages of medicine availability were computed along with standard errors.

As a second step, univariable and multivariable regression models were fitted to evaluate the individual and collective effects of the selected covariates on medicine availability across all dimensions. Linear, log-linear, and binomial models were fitted and compared based on the range of predictions and the distribution of residuals. Log-linear and binomial models provided little to no improvement compared to the linear model, so a linear model was chosen for its simplicity and versatility. Model diagnostics of the final model can be found in Supplementary Figs S1–S6.

A preselection of covariates included in the final model was made by fitting separate dimension-specific regression models for each dimension specified in Table 1. Covariates significant at the 5% level were included in a final full model. Collinearity between the remaining covariates was assessed using Pearson’s correlation coefficient. No strong correlations exceeding the threshold of ±0.8 were identified (Berry and Feldman 1985).

#### Analysis of explained variance of dimension-specific models

As a third step, we examined the explained variance (*R*^2^) of dimension-specific models (i.e. a model including covariates pertaining to one of the dimensions outlined in Table 1) and the relative change in *R*^2^ after removing each dimension from the full model. The first provides insight into how much variation in the availability of medicines is explained by each dimension. The latter provides insights into the contribution of each dimension to the full model. This step is essential to identify the key dimensions that drive variation in the availability of medicines.

#### Analysis of spatial autocorrelation

As a final step, we investigated the presence and strength of spatial autocorrelation in the data. The primary objective of this step was to evaluate whether the assumption of independent observations was violated. A secondary objective was to determine the spatial scale at which spatial autocorrelation exists and how much the model accounts for the observed spatial autocorrelation. As such, we quantified the presence and strength of spatial autocorrelation for (I) the availability of medicines; (II) the residuals of the full model; (III) the residuals of the full model, including a spatial lag; and (IV) the residuals of a univariable model with the province. Inverse distance weights were used to quantify the spatial relationship between healthcare facilities. Moran’s I was calculated to assess the level of spatial autocorrelation on distance bands ranging from 10 to 100 km, with 10-km increments. To address residual spatial autocorrelation, we conducted a sensitivity analysis by adding a spatial lag of the outcome variable based on the four nearest neighbours to the full model.

## Results

### Descriptive analysis of the availability of medicines

**Figure 1.**
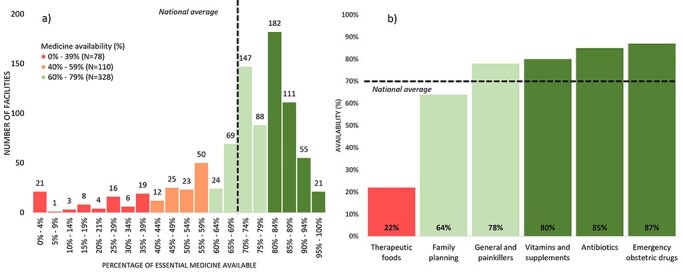
(a) Availability of medicines (%) across facility. (b) Availability of medicines (%) by type of medicine, 2021.

A total of 885 healthcare facilities were included in the study, of which 400 were SHCs (45%), 302 were BHCs (34%), 152 were CHCs (17%), and 31 were DHs (3%). Medicine availability varies from 0% to 100% between healthcare facilities, with averages of 70% (SE: 0.8%) nationally (Fig. 1a). Medicine availability varies considerably across different types of medicines (Fig. 1b). At least 80% of the required vitamins and supplements, antibiotics, and emergency obstetric care drugs were available on average, contrasting with therapeutic foods (22%), family planning methods (64%), and general painkillers (78%), which fell below the 80% threshold.

**Figure 2.**
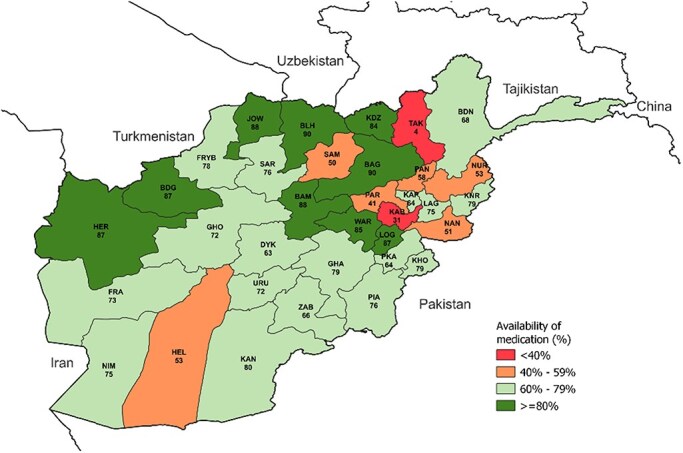
Availability of medicines (%) by province, Afghanistan, 2021.

Figure 2 shows the variation in the availability of medicines by province, which ranges from 4% in Takhar (SE: 1.7%) to 88% in Jawzjan (SE: 0.7%) and Bamyan (SE: 0.6%). Only nine provinces achieved an average availability of medicines of at least 80% (Badghis, Baghlan, Balkh, Bamyan, Herat, Jawzjan, Kunduz, Logar, and Wardak). The availability of medicines appears to be lower in Central and Eastern provinces and higher in Northern and South-eastern provinces.

### Regression analysis of the availability of medicines

This section describes the findings from the regression analysis outlined in Table 2. Unless otherwise stated, we will focus on multivariable regression results.

**Table 2.** Univariable and multivariable associations with the availability of medicines

			Univariable		Multivariable	
Covariate	N^a^	Medicine availability (%)	B	CI	B	CI
Intercept					68.4***	57.0–79.7
Sociopolitical						
District control^c^						
Government controlled (ref.)	331	68				
Taliban controlled	176	74	5.6**	2.2–9.1	3.1*	0.1–6.2^b^
Contested	378	70	1.5	−2.9 to 5.9	3.5*	0.7–6.2^b^
Taliban influence^d^						
No influence (ref.)	65	74				
Moderate influence	17	50	−24.3***	−30.5 to −18.1	1.2	−11.7 to 14.1
Medium influence	162	71	−3.9	−8.2 to 0.5	−1.7	−8.3 to 4.9
High influence	640	69	−4.5*	−8.1 to −0.9	−10.2**	−17.9 to −2.5^b^
Security incidents (#)^c^			0.1**	0.0–0.1	0.0**	0.0–0.1^b^
Civilian fatalities (#)^c^			−0.1	−0.2 to 0.1	−0.2*	−0.3 to −0.0
Geographical barriers						
Travel time to the provincial centre						
<4 h (ref.)	673	70				
4–8 h	135	75	5.7**	2.0–9.3	3.6*	0.5–6.6
>8 h	77	64	−5.8	−17.1 to 5.6	−1.6	−7.0 to 3.8
Travel time to the ring road (h)			−0.7	−1.7 to −0.3	−0.2	−0.8 to 0.3
Altitude						
Near the sea level	96	79				
Low altitude	524	68	−11.4***	−16.6 to −6.1	−3.6	−7.6 to 0.3
Moderate altitude	245	72	−7.2*	−12.8 to −1.7	−6.7	−11.2 to −2.2
High altitude	20	52	−26.9	−55.6 to 1.8	−26.7**	−39.1 to −14.4
Drought risk^c^	210	71	1.5	−1.2 to 4.2	8.6***	4.8–12.4
Avalanche risk^c^	81	60	−11.0*	−20.9 to −1.1	−15.6***	−20.6 to −16.3
Earthquake risk^c^	155	66	−4.5**	−7.5 to −1.4	−8.6***	−12.1 to −5.1
Landslide risk^c^	130	73	3.6	−3.9 to 11.1	−1.3	−8.9 to 6.3
Flood risk^c^	136	66	−5.0**	−8.4 to −1.5	−8.7**	−12.8 to −4.5
Managing pharmaceutical product supply						
Drug inventory available	742	73	17.1***	11.5–22.6	5.8**	2.0–9.7
Updated facility status report	810	72	19.4***	11.5–27.2	2.3	−2.7 to 7.2
Healthcare worker trained in pharmacy management	200	82	15.7***	12.8–18.6	0.9	−1.3 to 3.2
Level of satisfaction with availability of medicine^d^			0.0***	0.0–0.0	0.2***	0.0–0.4^b^
Ease of obtaining medicines	599	76	14.3***	10.7–17.9	5.2***	2.4–7.9
Local governance and stewardship						
National monitoring checklist	507	79	20.3***	17.0–23.6	6.1***	3.0–9.2
Supervision from provincial health directorate or NGO	770	72	19.1***	14.2–24.0	−1.6	−7.2 to 4.0
Securing and allocating funds						
Percentage of out-of-pocket expenditure spent on drugs^d^			−0.3***	−0.3 to −0.2	0.0	−0.1 to 0.1
Poverty^d^			0.0	−0.1 to 0.1	−0.1**	−0.2 to −0.0
Percentage of the contract value as lump sum^d^			0.1**	0.0–0.2	0.2***	0.1–0.3^b^
Budget per capita						
10–20 USD/cap (ref.)	335	67				
Contracting in	61	51	−20.3***	−25.8 to −14.9	−50.9***	−62.2 to −39.6
<10 USD/cap (ref.)	252	71	−4.4*	−8.4 to −0.4	−19.2***	−26.4 to −12.1
20–30 USD/cap	203	77	5.8**	2.3–9.3	−3.6	−7.5 to 0.2
>30 USD/cap	33	72	0.4	−3.7 to 4.5	−1.4	−6.7 to 4.0
Organizing and sustaining physical capacity						
Facility type						
SHC (ref.)	400	68				
BHC	302	68	0.6	−3.9 to 5.1	2.5*	0.6–4.5
CHC	152	77	9.4***	4.5–14.3	6.2***	3.8–8.6
DHs	31	79	11.3**	2.8–19.8	13.1***	9.1–17.2
Salary payment is not up-to-date	297	56	−20.6***	−24.0 to −17.3	−3.8**	−6.4 to −1.2
Healthcare worker to patient ratio (per 10 patients)			−2.3***	−3.1 to −1.6	−0.3	−1.0 to 0.5
Closed healthcare facility	14	29	−41.3***	−52.9 to −29.7	−6.2	−19.3 to 6.9
Guaranteeing product safety						
Electricity						
Electricity 24 h/day (ref.)	436	78				
Electricity most of the day	246	67	−10.6***	−14.5 to −6.6	−2.0	−4.9 to 0.9
Electricity is available a few hours per day	150	59	−18.5***	−24.4 to −12.6	−7.2***	−10.8 to −3.6
No electricity	53	52	−25.3***	−32.6 to −17.9	−4.3	−8.9 to 0.2
Pharmacy stock	713	73	12.1***	5.7–18.5	0.3	−2.4 to 3.0
Appropriate prescription practices	175	81	12.0***	8.4–15.6	0.2	−2.1 to 2.5

a Weighted numbers.

b The result is no longer significant after controlling for spatial autocorrelation.

c Measured at the district level.

d Measured at the provincial level.

* *P* < 0.05,

** *P* < 0.01.

*** *P* < 0.001.

#### Sociopolitical and geographical determinants

In 2021, specific districts were under Taliban control, others were under central government control, and some were contested (i.e. in dispute by government and antigovernment forces). Healthcare facilities in contested districts [regression coefficient *B*: 3.5, 95% confidence interval (CI): 0.7–6.2] are associated with slightly higher availability of medicines than those in government-controlled areas. Healthcare facilities located in a province with a high Taliban influence were associated with lower availability of medicines (*B*: −10.2, 95% CI: −17.9 to −2.5). Higher medicine availability was also associated with more security incidents (*B*: 0.0, 95% CI: 0.0–0.1) and fewer civilian fatalities (*B*: −0.2, 95% CI: −0.3 to −0.0), but these associations are weak.

Afghanistan is mountainous and prone to various natural hazards associated with the availability of medicine. Healthcare facilities located at higher altitudes (coefficients range from −3.6 at low altitudes to −26.7 at high altitudes) and healthcare facilities located in areas where there is a risk of avalanches, earthquakes, and flooding (coefficients range from −15.6 to −8.6) are associated with lower availability of medicines. In contrast, the risk of drought was found to be positively associated with medicine availability.

#### Elements of the local health and pharmaceutical systems

Improved capacities to manage pharmaceutical product supply were associated with a slight increase in medicine availability. For example, having a drug inventory was associated with a slight increase in medicine availability of 5.8 percentage points (95% CI: 2.0–9.7). Healthcare facilities in which at least 80% of clients were satisfied with the ease of obtaining medicines were associated with a slight increase in medicine availability of 5.2 percentage points (95% CI: 2.4–7.9).

We included two proxies to measure local governance and stewardship: application of the national monitoring checklist and supervision from the provincial health department or service provider. The MoPH developed the national monitoring checklist to regularly monitor the functionality of healthcare facilities. The application of the national monitoring checklist in healthcare facilities was associated with an increase in the availability of medicines by 6.1 percentage points (95% CI: 3.0–9.2). Supervision visits were associated with medicine availability in the univariable model (*B*: 19.1, 95% CI: 14.2–24.0) but not in the multivariable model.

The ability of local bodies and facilities to secure and allocate funds seems to contribute to improved medicine availability. The most important drivers were related to financial agreements with service providers. Service providers with a higher budget per capita and a higher lump sum (as a percentage of total contract value) were associated with increased availability of medicines (*B*: 0.2, 95% CI 0.1–0.3). Healthcare facilities managed by the MoPH under a contracting-in approach (i.e. in Panjsher, Parwan, and Kapisa) had far lower medicine availability than healthcare facilities managed by service providers (*B*: −50.9, 95% CI: −62.2 to −39.6).

Healthcare facilities with a greater ability to organize and sustain physical capacity had a higher availability of medicine. For instance, higher levels of primary healthcare facilities were associated with increased medicine availability. Conversely, healthcare facilities with delays in salary payments to healthcare workers had significantly lower medicine availability (*B*: −7.4, 95% CI: −10.1 to −4.6). Furthermore, the results suggest that a change in service providers contributed to the disruption of medicine procurement. In Takhar and Samangan provinces, several healthcare facilities were closed for some time before this study due to a change in service providers; these facilities were associated with a lower medicine availability of 6.2 percentage points.

The last function describing the local pharmaceutical system is ensuring product safety. We found that a lack of electricity was associated with lower medicine availability. Facilities with electricity available for most of the day or a few hours had a slightly, yet significantly, lower availability of medicines compared to facilities with electricity for 24 h per day.

### Analysis of explained variance (R^2^)

The full model, as presented in Table 2, explains 65% of the variation in medicine availability in Afghanistan’s public healthcare facilities, but not all model dimensions contribute equally to this (Table 3). Specifically, removing the dimensions of organizing and sustaining physical capacity, securing and allocating funds, and geographical barriers from the model, the *R*^2^ would drop by 6%, 14%, and 20%, respectively (Table 3). These results suggest that these three dimensions explain a considerable amount of variation not accounted for by other dimensions in the model. On the other hand, the dimensions of managing pharmaceutical product supply, local governance, and stewardship explain a substantial amount of variation (*R*^2^ > 20%) in a dimension-specific model (i.e. a model containing variables exclusively related to that dimension). However, their impact on the overall *R*^2^ is limited when removed from the full model.

**Table 3.** Overview of pseudo R^2^ for dimension-specific models and change in R^2^ after removing dimensions

	Dimension-specific model	Full model excluding dimension	
Dimension	*R* ^2^	*R* ^2^	% change
Insecurity	5%	63%	−3
Managing pharmaceutical product supply	24%	63%	−3
Local governance and stewardship	23%	64%	−2
Securing and allocating funds	24%	56%	−14
Organizing and sustaining physical capacity	28%	61%	−6
Guaranteeing product safety	19%	63%	−3
Geographical barriers	14%	52%	−20
*R* ^2^ of the full model	65%		

### Analysis of spatial autocorrelation

**Figure 3.**
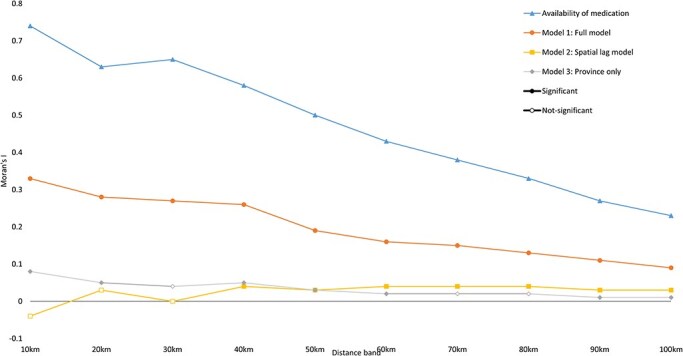
Spatial autocorrelation of availability of medicines and residuals of (1) full model residuals, (2) spatial lag model residuals, and (3) univariable model with province using inverse distance weights ranging from 10 to 100 km.

The results show a strong spatial clustering of the availability of medicines even at distances beyond 100 km (Fig. 3). The province appears to be the most effective predictor of medicine availability, explaining 78% of the variation between healthcare facilities. The model presented in Table 2 accounts for a considerable amount of the spatial autocorrelation but not all of it. This means that there are other processes not accounted for by the model that drive the remaining spatial autocorrelation. We ran a sensitivity analysis to account for the residual spatial autocorrelation by adding a spatially lagged variable (Supplementary Table S3). This model successfully accounts for the remaining spatial autocorrelation, but as a result, some associations presented in Table 2 are no longer significant. Covariates that are no longer significant after adding the spatial lag include conflict, security incidents, Taliban influence, level of satisfaction with ease of obtaining medicines, and percentage of contract values as a lump sum.

## Discussion

The study’s findings indicate that, despite facing significant sociopolitical challenges in 2021, Afghanistan’s health system managed to maintain a relatively sufficient supply of medicines in public healthcare facilities. Our findings suggest that geographical variations—rather than insecurity—posed significant barriers, emphasizing the need for targeted strategies in hard-to-reach areas. Contracting out healthcare services to nonstate service providers appears to contribute to improved availability of medicines. Nonetheless, certain aspects of the contracting design—particularly the ability to secure and allocate funds and the capacity to effectively manage and sustain physical resources—contributed to disparities in medicine availability between healthcare facilities.

The sociopolitical and geographical situation in Afghanistan makes healthcare service delivery challenging. Yet, despite these challenges, the average availability of 31 selected essential medicines was 70% in 2021 and 77% excluding therapeutic foods, which are not usually considered essential medicines in most countries. This is relatively high in comparison to other low- and middle-income countries such as Pakistan (35%), Indonesia (58%), India (50%), Lebanon (42%), and the Philippines (30%) (Saeed et al. 2019, Lambojon et al. 2020, Mohamed Ibrahim et al. 2021, Auton 2022, Fanda et al. 2024). However, we observed considerable geographical variation in the availability of medicines, ranging from 90% in Baghlan to 4% in Takhar. The low availability of therapeutic foods across the country is particularly concerning given the countries’ high level of malnutrition and food insecurity (Afghanistan Health Survey (AHS) 2018).

The study results suggest that insecurity was not a key factor driving variation in medicine availability in 2021. At the time of data collection, the Taliban was the main insurgent antigovernment group, controlling ∼20% and contesting >50% of Afghanistan’s 399 districts (Mapping Taliban Control in Afghanistan, 2017). A study into the effectiveness of contracting out service delivery confirms that service providers consider insecurity a key barrier to effective service provision (Salehi et al. 2018). Nonetheless, the study reports negligible differences in medicine availability between government-controlled, Taliban-controlled, or contested districts. This supports a growing body of evidence that contracting out healthcare services to nonstate providers enables the provision of healthcare in conflict-affected settings where governments may not have the capacity or legitimacy to provide these (Palmer et al. 2006, Abramson 2011, Khalil 2013, Alonge et al. 2015).

Afghanistan’s challenging topography negatively affects medicine availability. Higher altitudes and risk of various natural hazards such as avalanches, earthquakes, and flooding were associated with lower availability of medicines. These factors explain a considerable amount of variation in medicine availability. Indeed, service providers struggle to supply hard-to-reach areas with essential medical resources (Salehi et al. 2018). Data for this study were collected during spring months; during winter, parts of the country are inaccessible. The availability of medicines is likely lower during these months. Medicine shortages in hard-to-reach areas are of particular concern. Stock outs may persist longer due to logistical challenges, and the consequences for patients—who may have limited alternatives or need to travel further to obtain medication from other sources—could be more severe. Additional attention should be given to facilities in hard-to-reach areas to avoid stock outs. Strategies to address medicine shortages in these areas may include overstocking of priority medicines, improving data systems to monitor inventories, and implementing redistribution mechanisms (Iyengar et al. 2016, Zakumumpa et al. 2019).

The availability of medicines was considerably higher in provinces where services were contracted out to service providers than in provinces where services were contracted in to provincial health departments. A previous study confirms that the three contracting-in provinces (Panjsher, Parwan, and Kapisa), while experiencing advantages in security and logistics, were underperforming compared to contracting-out provinces (Salehi and Akseer). There are two potential explanations for this. First, contracting out provinces risked losing payments or contracts based on poor performance. As such, they had a higher incentive to improve medicine availability than contracting-in provinces, which were not penalized for poor performance. Second, the government centrally organized medicine procurement in the contracting-in provinces, which were prone to bureaucracy and delays (Blaakman and Lwin 2013). As suggested by Nepomnyashchiy and Yadav in their study in 2022, decentralizing medicine procurement can improve the availability of medicines at the healthcare facility level (Nepomnyashchiy and Yadav 2022).

However, the study findings also indicate that medicine availability is affected by the financial agreements made between service providers and the MoPH. Contracts were awarded based on quality and cost, incentivizing service providers to prepare a financially attractive offer (Salehi and Akseer). Indeed, we find that a lower cost per capita and a lower lump-sum budget were associated with lower medicine availability. The latter posed two risks for service providers: (I) losing funds due to unsatisfactory performance and (II) delays in payment due to delays in performance assessment. In line with the study findings, semiannual performance reviews by the MoPH in 2021 reported that low lump-sum budgets contributed to delays in staff salary payments for some provinces (Sehatmandi Provincial Semi-Annual Performance Review, 2019). Ensuring timely payment of funds, a minimum cost per capita, and a minimum lump-sum budget could guarantee a more stable cash flow for service providers required to procure medical supplies and pay staff salaries.

Facilities offering a higher level of service, such as CHCs and DHs, demonstrated a higher availability of medicines. Typically, these facilities are more extensive and provide a broader spectrum of services, thereby possessing a greater capacity to manage and organize physical resources. They also have access to improved storage facilities and are more likely to have a skilled pharmacist capable of accurately assessing the required quantity of medicines. Additionally, these facilities are generally located in more accessible and populated areas. Studies conducted in Ghana, Kenya, and Uganda have also indicated that facilities equipped with laboratories or vehicles (indicative of a higher level of care) or those at a higher service level experience lower levels of medicine stock outs (Masters et al. 2014, Okello et al. 2015). In Afghanistan, SHCs constitute a significant proportion of all primary healthcare facilities (World Health Organization—Regional Office for the Eastern Mediterranean). Enhancing medicine availability in these centres by, e.g., training healthcare workers on the quantification of medicines could substantially enhance population access to essential medicines.

One finding of particular concern is that healthcare facilities in provinces with a poorer population were associated with lower medicine availability. People from lower wealth quintiles are often disadvantaged regarding health outcomes and access to healthcare services (Akseer et al. 2016). When medicines are unavailable in healthcare facilities, they may turn to unlicensed vendors or private practitioners. As a result, they risk incurring higher out-of-pocket expenditures and exposure to substandard or falsified medicines (Hasnida et al. 2021, Nistor et al. 2023). The BPHS is already considered limited in scope. At a minimum, the resources to provide these services should be available nationwide.

The study’s most important strength lies in the quantity and comprehensiveness of the data, which allows us to study the availability of medicines in depth. However, there are some limitations worth highlighting. Most importantly, the study may overestimate the availability of medicine. This healthcare facility assessment was part of the performance assessment of service providers, creating perverse incentives for service providers and monitors to manipulate results (Alba et al. 2023). In addition, excluding medicines relevant to the treatment of leishmaniasis, tuberculosis, and malaria could bias the estimated medicine availability but allow for comparable estimates between facility types and provinces and avoid underestimating medicine availability in facilities that do not require them.

Furthermore, there are some statistical limitations pertaining to the persistent spatial autocorrelation of the model residuals. Adjusting the model for spatial autocorrelation by adding a spatially lagged variable mitigates this issue. Still, it may obscure relevant insights into the underlying processes that drive the spatial patterns under investigation. Despite the residual spatial autocorrelation, we maintain that the model provides insights into these underlying processes, contributing to our understanding of the spatial variation in medicine availability in Afghanistan. Additional model diagnostics based on the residuals (Supplementary Figs S1–S6) show a reasonable fit of the model.

Finally, the methods to measure medicine availability deviate from the standard method developed by the World Health Organization and Health Action International, as this study was conducted within a broader scope of monitoring and evaluation of public healthcare facilities. This assessment—including the methods to measure medicine availability and the selection of medicines—was designed in collaboration with NGOs, MoPH, and development partners (Peters et al. 2007). The estimated medicine availability of 31 selected medicines cannot be generalized to all essential medicines.

Future research should consider studying fluctuations in medicine availability and quality. Medicines are typically procured and supplied quarterly, and their availability at the quarter’s end can differ significantly from the beginning. In addition, Afghanistan’s challenges in regulating and ensuring the quality of imported drugs have been documented in previous research (Michael et al. 2013, Roien et al. 2022). Various studies and reports highlighted the presence of substandard or falsified medicines in public and private healthcare facilities (Michael et al. 2013, Lalani et al. 2015, Evaluation of Medicine Local Production vs. Importation in Afghanistan, 2020). Therefore, efforts to address medicine stock outs must include strategies to ensure medicine quality.

Our study analysed data from an assessment conducted before the Taliban takeover in August 2021. Yet, the analysis relating to medicine availability and the structure of Afghanistan’s health system remains relevant despite recent political changes. However, it is important to note that healthcare facility assessments conducted after 2021 indicate that the availability of medicine is yet to reach the levels documented in this study. In addition, since 2021, various changes have been implemented affecting contract structure and management and approaches to medicine procurement. However, our findings suggest that a decentralized procurement approach through service providers may contribute to essential medicine availability in Afghanistan. This underscores the importance for the MoPH and development partners to continue exploring procurement models that combine centralized quality control with decentralized distribution to enhance flexibility and accessibility, targeting lower-level healthcare facilities, and ensuring timely payment to service providers to cover running costs.

Lastly, contracting out healthcare services to nonstate providers is a common service delivery model in fragile and conflict-affected settings. Findings from this study that might be relevant for these countries include the decentralization of medicine procurement and regular monitoring of medicine availability as a means to improve medicine availability. When contracts are performance-based, donors and contract managers should ensure that budgets are realistic and paid timely.

## Conclusion

Despite facing substantial sociopolitical challenges in 2021, Afghanistan’s public health system maintained a satisfactory medicine supply, with an average availability of 70%. While the system exhibited resilience in areas with various levels of insecurity, geographical challenges posed an essential barrier to ensuring the supply of medicines, emphasizing the need for targeted strategies in hard-to-reach areas. Contracting out healthcare services to nonstate providers proved to be beneficial, yet systemic issues, such as fund allocation and resource management, contributed to disparities in medicine availability between facilities. In Afghanistan and other countries where healthcare services are contracted out, donors and contract managers have to ensure timely payments and adequate budgets for maintaining a proper supply of medical resources including essential medicines. In order to contribute to universal health coverage, policymakers should design strategies to improve medicine availability targeting facilities from lower service levels and areas with higher poverty levels.

## Supplementary Material

czae121_Supp

## Data Availability

Data may be obtained from a third party and are not publicly available.

## Reflexivity statement

This research was conducted by nine researchers, four of whom are women (including the first and second authors) and five are men. Three researchers are in the early stages of their career, and four researchers are from Afghanistan. The group consists of researchers with various backgrounds, including statistics and epidemiology, health and pharmaceutical systems, pharmaceutical sciences, and Afghanistan’s healthcare system. Their diversity in background led to this statistical approach to a health systems research question.
