# Supplementary material for: The availability of essential medicines in public healthcare facilities in Afghanistan: navigating sociopolitical and geographical challenges
Source: Health Policy Plan. 2024 Dec 19;40(3):368–79. doi: 10.1093/heapol/czae121 (PMC11886856; doi:10.1093/heapol/czae121)
Supplement: czae121_Supp [file czae121_supp.zip › Supplementary materials.docx]

**Supplementary materials**

**Supplementary Table 1. Covariates considered for analysis**

| **Dimension** | **Variable** | **Definition** |
| --- | --- | --- |
| **Security** | Control of district^a^ | Whether district is under government control, Taliban control, or contested on 9 July, 2021. |
|  | Taliban influence^b^ | Whether province is under no, moderate, medium or high Taliban influence. |
|  | Security incidents^a^ | Number of security incidents in a district from January until July 2021, including abduction/forced disappearance, air/drone strike, armed clash, attack, grenade, remote explosive/landmine/IED, shelling/artillery/missile attack, and suicide bomb. |
|  | Civilian fatalities^a^ | Number of civilians killed or injured resulting directly or indirectly from conflict-related violence between January and July 2021 in a district. |
| **Local governance and stewardship** | National Monitoring Checklist | The facility applied a national monitoring checklist at least once in the past 12 months. |
| **Organizing and sustaining physical and human resources** | Facility type | Type of facility (sub-health center, basic health center, comprehensive health center, or district hospital). |
|  | Salary payment up-to-date | At least one health worker reports that salary payment is not up-to-date. |
|  | Health worker-to-patient ratio | Number of health workers per 100 patients. |
|  | Change in NGO^b^ | Whether or not there has been a change in implementing NGOs as of January 2019. |
|  | Closed health facility | The facility was temporarily closed in the month preceding the survey. |
| **Managing pharmaceutical product supply** | Drug inventory | The facility has a drug inventory. |
|  | Patient satisfaction with ease of obtaining medicine | At least 80% of clients are satisfied with the ease of obtaining medicines. |
|  | Pharmacy management training | At least one health worker trained in pharmacy management in the past 12 months. |
|  | Patient satisfaction with availability of medicines^b^ | The average level of satisfaction with the availability of medicines in the province. |
| **Securing and allocating funds** | Managing agency | The facility is managed by MoPH without support, MoPH with support, or NGO. |
|  | Out-of-pocket expenditure^b^ | Provincial average OOPE for out-patient visits per 100 Afghani. |
|  | Out-of-pocket expenditure on drugs^b^ | Share of provincial average of OOPE for out-patient visits spend on medicines. |
|  | Petty cash system | A petty cash system is available, including petty cash and expenditure records. |
| **Guaranteeing product safety** | Electricity supply | Electricity is available all day, most of the day, a few hours per day, or not at all. |
|  | Appropriate prescription practices | There is evidence that health workers give out medicines without proper instructions. |
|  | Pharmacy stock | Pharmacy stock (including thermometer and exhaust fan) is present and functional. |
|  | Expired products | The facility has expired pharmaceuticals or pharmaceuticals without readable expiry dates in stock. |
| **Geography** | Travel time to the provincial center | Travel time from the health facility to the provincial center by car is less than 4 hours, between 4 and 8 hours, or more than 8 hours. |
|  | Altitude | Health facility located on sea level (<500m), low altitude (500m – 2000m), moderate altitude (2000m – 3000m) or high altitude (>3000m). |
|  | Border district | The facility is located in a district with an international border. |
|  | Risk of natural hazards | The facility is located in a district at high risk of natural hazards such as avalanches, earthquakes, landslides, flooding, or drought. |
| **Capacity and demand** | Average daily patient load | Average daily patient load in the past six months. |
|  | Coverage of skilled birth attendance^b^ | Percentage of women with a live birth in 2 years preceding the survey who received skilled birth attendance. |
|  | Sehatmandi facilities | Percentage of Sehatmandi facilities out of all public health facilities. |
|  | Population density^a^ | Number of people per square kilometer. |

**Supplementary Table 2 Essential medicines assessed**

| **Indicator 11** | **Pharmaceuticals and Vaccines Availability Index** |
| --- | --- |
| **General and painkillers** | Tetracycline ophthalmic ointment |
|  | Paracetamol tabs |
|  | Amoxicillin / Ampicillin (syrup, tabs, or capsule) |
|  | ORS packets |
|  | Iron tabs (with or without folic acid) |
|  | Iron syrup |
|  | Cotrimoxazole |
|  | Salbutamol |
|  | Adrenaline |
| **Vitamins and supplements** | Folic Acid |
|  | Mebendazole |
|  | Metronidazole |
|  | Sodium Lactate |
|  | Iodine |
|  | Retinol (Vitamin A) |
|  | Zinc |
|  | Vitamin K |
|  | Chlorhexidine |
| **Therapeutic foods** | RUTF |
|  | F-75 |
|  | F-100 |
|  | ReSoMal |
| **Contraceptives** | Condoms |
|  | Oral contraceptive tablets (COC) |
|  | Oral contraceptive tablets (POP) |
|  | DMPA-IM |
|  | DMPA-SC |
|  | IUD |
|  | Implant |
| **Emergency obstetric drugs** | Magnesium Sulfate |
|  | Oxytocin |

**Supplementary figures 1-6 Model diagnostics**

*Full model without spatial lag*

Figure 1. Residuals versus predicted values Figure 2. Histogram of residuals


Figure 3. Q-Q plot of model residuals

*Full model with spatial lag*

Figure 4. Residuals versus predicted values Figure 5. Histogram of model residuals

Figure 6. Q-Q plot of model residuals

**

**Supplementary Table 3 Results of multivariable analysis with spatial lag**

| **Covariate** | **Multivariable** | |
| --- | --- | --- |
|  | **B** | **CI** |
| Intercept | 25·4*** | [12·0 – 38·8] |
| Spatial lag (4 nearest neighbors) | 60·1 | [49·5 – 70·6] |
| *Socio-political* |  |  |
| District control^a^ |  |  |
| Govt controlled (ref·) |  |  |
| Taliban controlled | 0·3 | [-2·5 – 3·1] |
| Contested | 1·2 | [-1·2 – 3·7] |
| Taliban influence^b^ |  |  |
| No influence (ref·) |  |  |
| Moderate influence | -0·8 | [-12·3 – 10·7] |
| Medium influence | -0·2 | [-6·2 – 5·7] |
| High influence | -5·8 | [-12·7 – 1·2] |
| Security incidents (#)^a^ | 0·0 | [-0·0 - 0·1] |
| Civilian fatalities (#)^a^ | -0·1* | [-0·2 - -0·0] |
| *Geographical barriers* |  |  |
| Travel time to the provincial center |  |  |
| Less than 4hr (ref·) |  |  |
| 4 to 8 hours | 3·6* | [0·5 - 6·6] |
| More than 8 hours | -1·6 | [-7·0 – 3·8] |
| Travel time to ring road (hr·) | -0·2 | [-0·8 – 0·3] |
| Altitude |  |  |
| Near sea level |  |  |
| Low altitude | -3·6 | [-7·6 – 0·3] |
| Moderate altitude | -6·7 | [-11·2 - -2·2] |
| High altitude | -26·7** | [-39·1 - -14·4] |
| Drought risk^a^ | 4·3*** | [1·1 – 7·4] |
| Avalanche risk^a^ | -19·4*** | [-14·6 - -4·3] |
| Earthquake risk^a^ | -2·8*** | [-5·8 – 0·1] |
| Landslide risk^a^ | 0·1 | [-6·8 – 6·9] |
| Flood risk^a^ | -6·1** | [-9·9 - -2·3] |
| *Managing pharmaceutical product supply* |  |  |
| Drug inventory available | 5·2** | [1·7 - 8·7] |
| Updated FSR | 2·1 | [-2·5 – 6·7] |
| HW trained in pharmacy management | -1·6 | [-4·0 – 0·8] |
| Level of satisfaction with  availability of medicine^b^ | 0·0 | [-0·1 - 0·2] |
| Ease of obtaining medicines | 3·1* | [0·6 – 5·5] |
| *Local governance and stewardship* |  |  |
| National monitoring checklist | 3·7*** | [1·1 – 6·4] |
| Supervision from PHD or NGO | -0·8 | [-5·3 – 3·8] |
| *Securing and allocating funds* |  |  |
| % of OOPE spend on drugs^b^ | 0·0 | [-0·1 - 0·1] |
| Poverty ^b^ | -0·1** | [-0·2 - -0·0] |
| % of the contract value as lump sum^b^ | 0·0 | [-0·0 - 0·1] |
| Budget per capita |  |  |
| 10-20 USD/cap (ref·) |  |  |
| Contracting in | -22*** | [-33·5 - -11·1] |
| <10 USD/cap (ref·) | -9·7*** | [-15·2 - -4·1] |
| 20-30 USD/cap | -1·6 | [-5·1 – 1·9] |
| >30 USD/cap | 0·3 | [-4·2 - 4·8] |
| *Organizing and sustaining physical capacity* |  |  |
| Facility type |  |  |
| Sub-health center (ref·) |  |  |
| Basic health center | 2·5* | [0·6 - 4·5] |
| Comprehensive health center | 6·2*** | [3·8 - 8·6] |
| District hospitals | 13·1*** | [9·1 - 17·2] |
| Salary payment is not up-to-date | -3·8** | [-6·4 - -1·2] |
| HW to patient ratio (per 10 patients) | -0·3 | [-1·0 - 0·5] |
| Closed HF | -6·2 | [-19·3 - 6·9] |
| *Guaranteeing product safety* |  |  |
| Electricity |  |  |
| Electricity 24h/day (ref·) |  |  |
| Electricity most of the day | -2·0 | [-4·9 - 0·9] |
| Electricity is available few hours per day | -7·2*** | [-10·8 - -3·6] |
| No electricity | -4·3 | [-8·9 - 0·2] |
| Pharmacy stock | 0·3 | [-2·4 - 3·0] |
| Appropriate prescription practices | 0·2 | [-2·1 - 2·5] |

**Supplementary Table 4 Missing data per variable**

| **Covariate** |  |
| --- | --- |
|  | **# missing (%)** |
| Intercept |  |
| *Security (# missing; %)* |  |
| District control | 0 (0%) |
| Taliban influenceb | 0 (0%) |
| Security incidents (#) | 0 (0%) |
| Civilian fatalities (#) | 0 (0%) |
| *Managing pharmaceutical product supply* |  |
| Drug inventory available | 1 (0%) |
| Updated FSR | 1 (0%) |
| HW trained in pharmacy management | 1 (0%) |
| Level of satisfaction with availability of medicine^b^ | 0 (0%) |
| Ease of obtaining medication | 20 (2%) |
| *Local governance and stewardship* |  |
| National monitoring checklist | 0 (0%) |
| Supervision from PHD or NGO | 0 (0%) |
| *Securing and allocating funds* |  |
| % of OOPE spend on drugsb | 0 (0%) |
| % of people in lowest wealth quintile | 0 (0%) |
| % of contract value as lumpsum | 0 (0%) |
| Budget per capita | 0 (0%) |
| *Organizing and sustaining physical capacity* |  |
| Facility type | 0 (0%) |
| Salary payment not up-to-date | 1 (0%) |
| HW to patient ratio (per 10 patients) | 1 (0%) |
| Closed HF | 0 (0%) |
| *Guarenteeing product safety* |  |
| Electricity | 0 (0%) |
| Pharmacy stock | 2 (0%) |
| Appropriate prescription practices | 20 (2%) |
| *Geographical barriers* |  |
| Travel time to provincial center | 0 (0%) |
| Altitude | 0 (0%) |
| Drought risk | 0 (0%) |
| Avalanche risk | 0 (0%) |
| Earthquake risk | 0 (0%) |
| Landslide risk | 0 (0%) |
| Flood risk | 0 (0%) |
| Time to ringroad | 0 (0%) |
